# Mutagenesis facilitated crystallization of GLP-1R

**DOI:** 10.1107/S2052252519013496

**Published:** 2019-10-17

**Authors:** Yueming Xu, Yuxia Wang, Yang Wang, Kaiwen Liu, Yao Peng, Deqiang Yao, Houchao Tao, Haiguang Liu, Gaojie Song

**Affiliations:** a Shanghai Key Laboratory of Regulatory Biology, Institute of Biomedical Sciences and School of Life Sciences, East China Normal University, Shanghai 200241, People’s Republic of China; b iHuman Institute, ShanghaiTech University, 393 Middle Huaxia Road, Shanghai 201210, People’s Republic of China; c Complex Systems Division, Beijing Computational Science Research Center, Beijing 100193, People’s Republic of China; d School of Life Science and Technology, ShanghaiTech University, Shanghai 201210, People’s Republic of China

**Keywords:** mutations, G-protein-coupled receptors, glucagon-like peptide-1 receptor, membrane proteins, molecular dynamic simulations, crystallization

## Abstract

The class B family of G-protein-coupled receptors (GPCRs) has long been a paradigm for peptide hormone recognition and signal transduction. One class B GPCR, the glucagon-like peptide-1 receptor (GLP-1R), has been considered as an anti-diabetes drug target and there are several peptidic drugs available for the treatment of this overwhelming disease. The previously determined structures of inactive GLP-1R in complex with two negative allosteric modulators include ten thermal-stabilizing mutations that were selected from a total of 98 designed mutations. Here we systematically summarize all 98 mutations we have tested and the results suggest that the mutagenesis strategy that strengthens inter-helical hydro­phobic interactions shows the highest success rate. We further investigate four back mutations by thermal-shift assay, crystallization and molecular dynamic simulations, and conclude that mutation I196^2.66b^F increases thermal stability intrinsically and that mutation S271^4.47b^A decreases crystal packing entropy extrinsically, while mutations S193^2.63b^C and M233^3.36b^C may be dispensable since these two cysteines are not di­sulfide-linked. Our results indicate intrinsic connections between different regions of GPCR transmembrane helices and the current data suggest a general mutagenesis principle for structural determination of GPCRs and other membrane proteins.

## Abstract 2

The ∼100 mutations used during the structural determination of GLP-1R suggest multiple mutagenesis strategies, which may find applications in the crystallization of G-protein-coupled receptors and other membrane proteins, and potentially in functional and pharmacological studies by locking proteins into specific conformations.

## 1. Introduction   

Type 2 diabetes is a long-term metabolic disorder that is predicted to affect ∼10% of the adult population by 2030 (Shaw *et al.*, 2010 ▸). Major causes of type 2 diabetes are a lack of insulin or insulin resistance, both of which cause high blood sugar. Current treatments of type 2 diabetes include injection of insulin or peptide agonists of glucagon-like peptide-1 receptor (GLP-1R) that provoke the synthesis and release of insulin (Gutniak *et al.*, 1992 ▸). GLP-1R belongs to the class B family of G-protein-coupled receptors (GPCRs), a group characterized by a 120–160 residue folded extracellular domain (ECD) followed by a canonical transmembrane domain (TMD) (Graaf *et al.*, 2016 ▸). The widely accepted two-domain binding model suggests the ECDs of class B receptors recognize the C-terminal helices of their hormone peptide ligands, thus facilitating binding of the N-terminal region of the peptides to the TMDs for downstream signaling (Hoare, 2005 ▸). Currently, discovery of anti-diabetes drugs is limited to peptide agonists including GLP-1, extendin-4 and their analogs (Pabreja *et al.*, 2014 ▸), while previously the development of small-molecule drugs to target this receptor was extremely challenging because of the lack of structural information about druggable small-molecule binding sites.

Two TMD structures of class B receptors [corticotropin-releasing factor 1 (CRF1R) and glucagon receptor (GCGR)] (Hollenstein *et al.*, 2013 ▸; Siu *et al.*, 2013 ▸) were solved in 2013 and these structures revealed interesting diversities within this subfamily. The GCGR structure features an extraordinary extended helix (called the stalk region) at the N-terminus of transmembrane helix 1 (TM1) and a tilted helix 8 region at the C-terminal end, whereas the crystallized CRF1R is truncated before helix 8 making it impossible to reveal its conformation. In 2016, a second structure of thermal-stabilized GCGR was solved but the construct lacked the C-terminal three-helical turns of helix 8 and the N-terminus of the stalk region in TM1 (Jazayeri *et al.*, 2016 ▸). The recently published crystal structures of GLP-1R TMD (Song *et al.*, 2017 ▸), full length GCGR (Zhang, Qiao *et al.*, 2017 ▸), full length GLP-1R (Jazayeri *et al.*, 2017 ▸), and cryoEM structures of G_s_-protein-bound GLP-1R (Zhang, Sun *et al.*, 2017 ▸), calcitonin receptor (CTR) (Shihoya *et al.*, 2016 ▸) and parathyroid hormone receptor 1 (PTH1R) (Zhao *et al.*, 2019 ▸) have extended our understanding of the structural determinants of class B GPCR function and modulation by small molecules, peptide ligands and functional antibodies.

In the past 12 years, the determination of more than 40 novel GPCR crystal structures has provided valuable information for understanding GPCR signaling and drug discovery (Thal *et al.*, 2018 ▸). However, crystallization is still hampered by the highly dynamic nature of GCPRs in solution and the existence of multiple metastable states. To enhance the stability of GPCRs, thermal-stabilizing mutations were introduced to the expression construct for crystallization. Structures solved by this approach include the β1-adrenergic receptor (Warne *et al.*, 2008 ▸), agonist-bound adenosine A_2A_ (Lebon & Tate, 2011 ▸), neurotensin receptor 1 (White *et al.*, 2012 ▸), metabotropic glutamate 5 (mGluR5) (Doré *et al.*, 2014 ▸), CRF1R (Hollenstein *et al.*, 2013 ▸), free fatty-acid receptor 1 (FFAR1, also known as GPR40) (Srivastava *et al.*, 2014 ▸), GCGR (Jazayeri *et al.*, 2016 ▸), CC chemokine receptor 9 (CCR9) (Oswald *et al.*, 2016 ▸), protease-activated receptor 2 (PAR2) (Cheng *et al.*, 2017 ▸), GLP-1R TMD (Song *et al.*, 2017 ▸) and the recently solved melatonin receptor 1 and 2 (Stauch *et al.*, 2019 ▸; Johansson *et al.*, 2019 ▸). Wild-type TMD of GLP-1R (residue range 128–431) is extremely unstable as suggested by size-exclusion chromatography (SEC) and thermal-shift assay (Song *et al.*, 2017 ▸). We have previously described that ten thermal-stabilizing mutations were introduced in the TMD structure of GLP-1R in complex with two negative allosteric modulators (NAMs), PF-06372222 and NNC0640, and we showed that a di­sulfide bond (I317^5.47b^C—G361^6.50b^C) and a GCGR mimicking mutation C347^6.36b^F are indispensable for crystallization (Song *et al.*, 2017 ▸). Here, we further summarize all of the 98 mutations that we have tested, analyze the effects of thermal-stabilizing mutations and describe attempts to crystallize several constructs with back mutations. Our experimental data combined with molecular dynamic (MD) simulations suggest general principles for mutagenesis design to increase thermal stability and crystallization success rates of GPCRs and other membrane proteins.

## 2. Results   

### 2.1. Mutation overview   

We built a model of GLP-1R based on a previously solved GCGR structure (PDB entry 4l6r; Siu *et al.*, 2013 ▸) that we used as a template for mutagenesis design to stabilize the transmembrane bundle, especially the thermodynamic regions revealed in the homologous GCGR structure (*e.g.* the extracellular halves of TM3–6). The principle for mutagenesis design was to strengthen inter-helical interaction patterns by predicting hydrogen bonds (salt bridges), hydrophobic interactions and di­sulfide bonds, or to strengthen ligand–receptor interaction patterns by covalent bonds or other interactions. Besides manual prediction based on modeling, the prediction of di­sulfide bonds was also assisted by a di­sulfide prediction algorithm (Pu *et al.*, 2018 ▸). In each round beneficial mutations were passed on to the next round after consideration of monodispersity (the percentage of monomeric fraction in total fractions in SEC), protein yield (the height of monomeric fraction in SEC) and thermal stability (the melting temperature). At earlier stages we mainly selected the mutations with improved homogeneities (using protein yield as another reference), while at later stages we mostly considered the thermal stabilities of mutations since monodispersities were already sufficiently high (see the Methods section[Sec sec4]).

Briefly, the results show that while most of these double-cysteine mutations were unfavorable in the constructs, we successfully screened two pairs (I317^5.47b^C—G361^6.50b^C, S193^2.63b^C—M233^3.36b^C) that significantly increased protein homogeneity [see Figs. S1(*a*), S1(*b*) and Table S1 in the Supporting information], correlating with the strict restriction of the Cβ–Cβ distance and dihedral angle for di­sulfide bonds. In contrast, most of the single point mutations showed moderate effects, and a relatively higher proportion of mutations increased protein homogeneity [Figs. S1(*c*)–S1(*e*) and Table S2]. Specifically, we observed eight single point mutations that aided protein homogeneity through hydrophobic interactions, whereas mutations by predicting inter-helical hydrogen bonds or ligand–receptor covalent bonds are yet to be successful. Actually, all six single point mutations in the final crystallization construct were mutated either from hydrophilic to hydrophobic residues (S225^3.28b^A, S271^4.47b^A, G318^5.48b^I, K346^6.35b^A), or from hydrophobic to bulky hydrophobic residues (I196^2.66b^F, C347^6.36b^F). The design rationale for these ten mutations is further analyzed below, while other mutations are summarized in Tables S1, S2 and Fig. S1.

### 2.2. Construct design   

**Figure 1:**
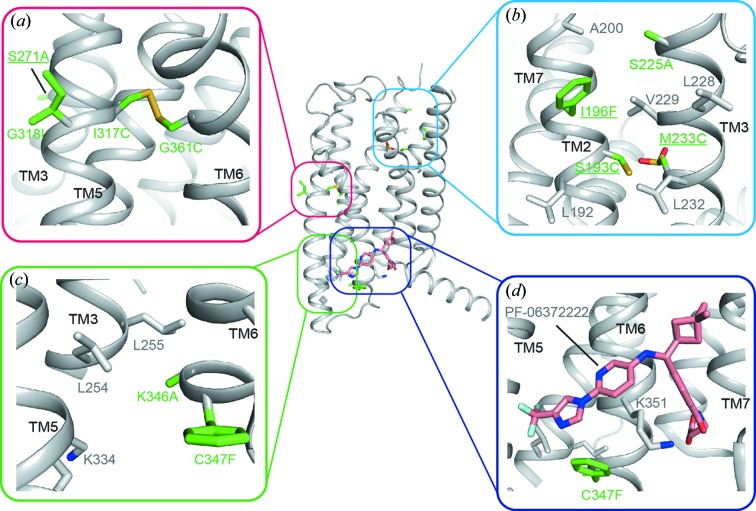
Locations of the ten thermal-stabilized mutations. (*a*)–(*d*) The original structure of inactive GLP-1R in complex with PF-06372222 (PDB entry 5vew; Song *et al.*, 2017 ▸) is shown as a gray cartoon, with mutations as green sticks and other interacting residues as gray sticks. Mutations analyzed in the current study are underlined.

Since glycine residues usually provide flexibility for conformational equilibrium of GPCRs, we mutated Gly318^5.48b^ to isoleucine (G318^5.48b^I) to facilitate crystallization based on sequence alignment within class B receptors [Fig. 1 ▸(*a*)]. Furthermore, Gly361^6.50b^ in TM6 was mutated to cysteine to form a di­sulfide bond with I317^5.47b^C for linking the middle region of TM5 and TM6, the most thermodynamic region in the previously solved GCGR structure [Fig. 1 ▸(*a*)]. The mutation S225^3.28b^A fitted the nearby hydrophobic environment provided by Ile196^2.66b^, Leu224^3.27b^, Leu228^3.31b^ and Val229^3.32b^, while I196^2.66b^F further stabilized the orientations of TM2 and TM3 by forming a patch of hydro­phobic interactions with TM3 residues [Fig. 1 ▸(*b*)]. Ser271^4.47b^ was mutated to alanine as it was facing the lipid bilayer [Fig. 1 ▸(*a*)]. Lys346^6.35b^ is located at the intracellular tip of TM6, and the K346^6.35b^A mutation makes it stay in a close position with the corresponding residues Leu254^3.57b^, Leu255^3.58b^ and Lys334^5.64b^ that mimic the inactive conformation of GPCRs [Fig. 1 ▸(*c*)]. C347^6.36b^F is a GCGR mimicking mutation used to strengthen the hydrophobic interaction with NAMs and Lys351^6.40b^ based on previous MK0893-bound GCGR structure [Fig. 1 ▸(*d*)]. Besides these two cysteine mutations that successfully formed a di­sulfide bond (I317^5.47b^C—G361^6.50b^C), our attempts to introduce another di­sulfide bond pair (S193^2.63b^C—M233^3.36b^C) was not successful according to their densities in the solved crystal structure. This finding indicated that these two cysteines may function independently to improve the monodispersity or thermal stability at that stage of construct optimization. Interestingly, in the solved structure we found that the Cys233^3.36b^ was oxidized into sulfinic acid (CSD233^3.36b^) during crystallization [Fig. 1 ▸(*b*)], a modification that was frequently observed, *e.g.* in the structure of *Medicago sativa* chalcone synthase (Ferrer *et al.*, 1999 ▸).

To study the effects of these sites we made further constructs in which we mutated back single point (C233^3.36b^M, named M9 hereafter), double points (C193^2.63b^S/C233^3.36b^M, named M8 hereafter) and quadruple points (C193^2.63b^S/C233^3.36b^M/A271^4.47b^S/F196^2.66b^I, named M6 hereafter). These mutants covered the sites where the engineered mutations seemed unnecessary since a di­sulfide bond was not formed (C193^2.63b^S/C233^3.36b^M), as well as the sites that affect either intramolecular interactions (F196^2.66b^I) or intermolecular interactions during crystal packing (A271^4.47b^S). These constructs were purified, characterized, crystallized and compared with the construct (named M10 hereafter) in the previously published crystal structure (Song *et al.*, 2017 ▸).

### 2.3. Thermal stability   

**Figure 2:**
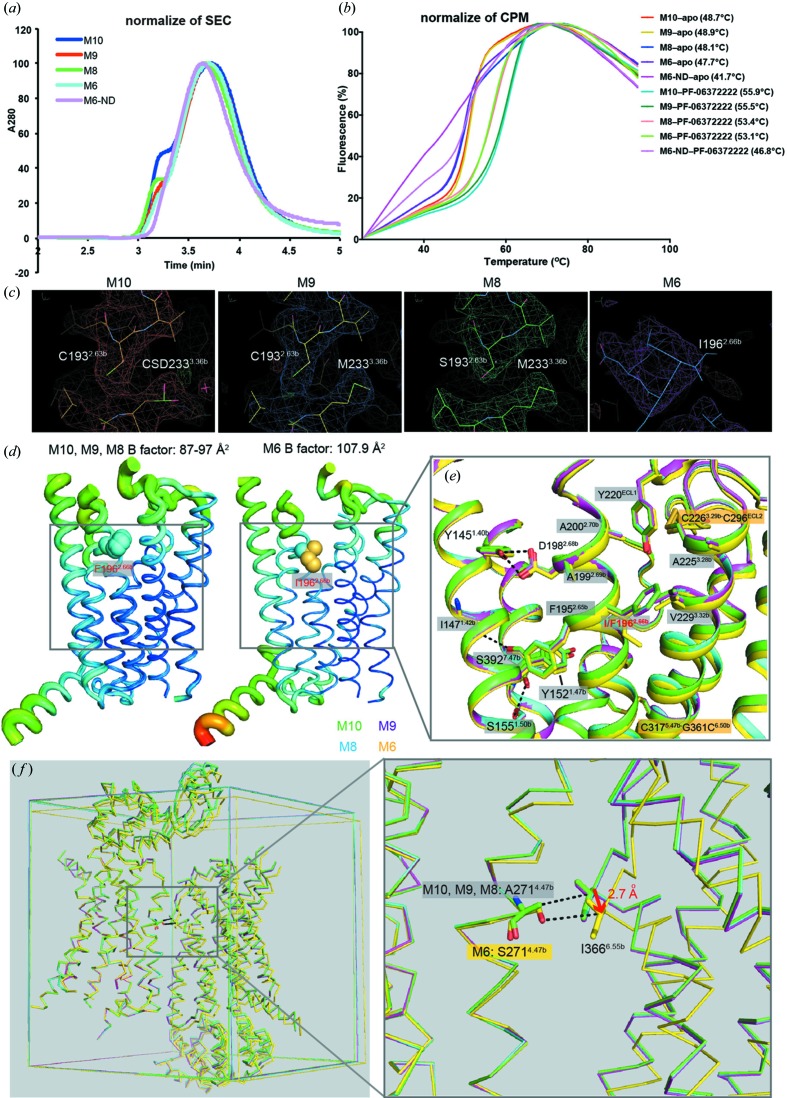
Thermal-shift assay of GLP-1R mutants, and potential mechanisms of I196^2.66b^F and S271^4.47b^A in the thermal stability and crystallization of GLP-1R. (*a*) SEC of GLP-1R fusion proteins suggests that the GLP-1R mutants are mostly monomeric and of similar homogeneity. (*b*) Thermal-shift assay of GLP-1R mutants in apo state or in complex with ligand PF-06372222. The different melting temperatures of the GLP-1R mutants indicating these mutations significantly affect thermal stability. (*c*) Densities of representative mutations in mutant structures. (*d*) *B* factor and distribution map of four constructs. (*e*) I196^2.66b^F mutation stabilized GLP-1R through its connections with the central polar network and other regions. (*f*) Packing and asymmetric unit of the four crystallized constructs. The back mutation of A271^4.47b^S induced significant change in the cell contents of M6 (yellow) compared with the other three back mutations and disturbed the packing. The four structures are superimposed through chain b. In Figs. 2 ▸(*e*) and 2 ▸(*f*) the color codes are shown in the key at the center.

All mutants were expressed in insect cells and purified to high homogeneity [Fig. 2 ▸(*a*)]. We then measured the thermal stabilities of these proteins in complex with PF-06372222 by a fluorescence-based thermal-shift assay [Fig. 2 ▸(*b*)]. As a control, we also measured the thermal-shift data of apo proteins whose melting temperatures decreased by 5–8°C, suggesting that the PF-06372222 indeed stabilized the receptor in each mutant. Fig. 2 ▸(*b*) shows that compared with M10, the M9 and M8 constructs have similar and slightly lower melting temperatures, respectively, which is consistent with our finding that residues Cys193^2.63b^ and Cys233^3.36b^ are not di­sulfide linked. In contrast, further removal of the other two cysteines, which are di­sulfide linked (C193^2.63b^S/C233^3.36b^M/C317^5.47b^I/C361^6.50b^G, named M6-ND hereafter), induced a decrease of ∼9°C in the melting temperature, and the M6-ND protein could not be crystallized in our crystallization trials. The similar melting temperatures of M9 and M10 suggest that the endogenous M233 residue and the modified cysteine residue (CSD233) contributed similarly to thermal stability, while the ∼2°C decrease of M8 and M6 indicates that the back mutations of C193^2.63b^S, A271^4.47b^S and F196^2.66b^I slightly affected the protein stability in solution.

### 2.4. Crystallization   

The three new constructs were crystallized in complex with PF-06372222 in the same condition as the M10 crystals (Fig. S2). GPCR crystals are prone to radiation damage and decay easily under a synchrotron X-ray source, and so one usually has to collect and merge data from many high-diffraction-quality crystals. Furthermore, the non-symmetric space group of *P*1 makes the data collection of GLP-1R crystals challenging and time consuming. While the previous M10 crystals were collected to a completeness of 95%, we could only collect data of the three new constructs to a relatively low level of completeness (M9, 88.8%; M8, 77.9% and M6, 79.9%) because of limited resources. Despite fewer measured crystals, the available data show comparable statistics with the M10 crystals, *e.g.* similar *R*_merge_ and *I*/σ values (Table 1 ▸). The new crystals were processed to the same space group and structures were determined by molecular replacement successfully. The densities of key side chains indicated successful mutagenesis of specified residues [Fig. 2 ▸(*c*)]. The cell contents of M9 and M8 are within 2% variation compared with M10, whereas the differences between M6 and the other three are larger. For example, the *b* axis of M6 (71.1 Å) is 4.7 Å longer than that of M10 (66.4 Å), while the *c* axis of M6 is 2.4 Å shorter (Table 1 ▸). The crystals of M9 and M8 were both processed to 2.8 Å resolutions and their structures were refined to an *R*_work_/*R*_free_ of 0.247/0.280 and 0.244/0.290, respectively. Notably, the M6 crystals were apparently worse than the others and the collected M6 data were cut to 3.1 Å using the same criteria (CC_1/2_ > 0.6). Indeed, our attempts to include higher resolution (<3.1 Å) data of M6 generated a bad density map and an even worse *R*_work_/*R*_free_. The 3.1 Å M6 structure was finally refined to 0.257 (*R*_work_) and 0.303 (*R*_free_); in line with this, the *B* factor of M6 (107.9 Å^2^) is also significantly higher than the other three crystallized constructs (87–97 Å^2^) [Table 1 ▸ and Fig. 2 ▸(*d*)].

Compared with M8, M6 shared a similar thermal stability but much worse crystal diffraction, which must be a consequence of the two back mutations in M6, F196^2.66b^I and A271^4.47b^S. Reflecting on the positions of these two residues, we reasoned that the mutations F196^2.66b^I and A271^4.47b^S affected intrinsic thermal stability and extrinsic crystal packing, respectively. Phe196^2.66b^ was located in the extracellular half of TM2, and the bulky phenyl group formed stronger hydrophobic interactions with nearby residues Tyr220^ECL1^, Ala225^3.28b^, Val229^3.32b^, Ala199^2.69b^ and Ala200^2.70b^ [Fig. 2 ▸(*e*)], thus the back mutation of F196^2.66b^I weakened the inter-helical interactions and thermal stability of the TM2–ECL1–TM3 region. In contrast, Ala271^4.47b^ sat in the interface of TM4 with the C-terminal tail of TM6 in the symmetry-related molecule [Fig. 2 ▸(*f*)]. Compared with alanine, the additional hydroxyl group of A271^4.47b^S pushed the symmetry-related molecule away mostly along the *b* axis, and the opposing residue Ile366^6.55b^ moved by 2.7 Å. This explained the apparent distinct cell contents of M6 crystals mentioned above. Moreover, the incompatibility between Ser271^4.47b^ and opposing hydrophobic residues including Ile366^6.55b^ generated high entropy that was unfavorable for crystal packing. Nevertheless, the effects of A271^4.47b^S in solution are very limited, as suggested by M6’s slightly decreased thermal stability compared with other mutants [Fig. 2 ▸(*b*)]. To study further the effects of these four mutations we conducted MD simulations to investigate the dynamics of GLP-1R mutants in aqueous environments.

### 2.5. MD simulations   

MD simulations were carried out to investigate the effects of mutations on the stability of the GLP-1R TMD. Since the fusion partner T4 lysozyme (T4L) was included in each expression construct for thermal-stability measurement, we also included T4L in the MD simulations so the results are comparable to each other. To perturb the system minimally, three new mutants were built based on the M10 structure and the rotamers of these mutated residues were chosen based on the densities in their corresponding crystal structures (M9, M8 and M6). In the M10 structure, the special residue CSD233^3.36b^, whose force field parameters were not available in the database of CHARMM36, was substituted by wild-type cysteine residue during simulations.

**Figure 3:**
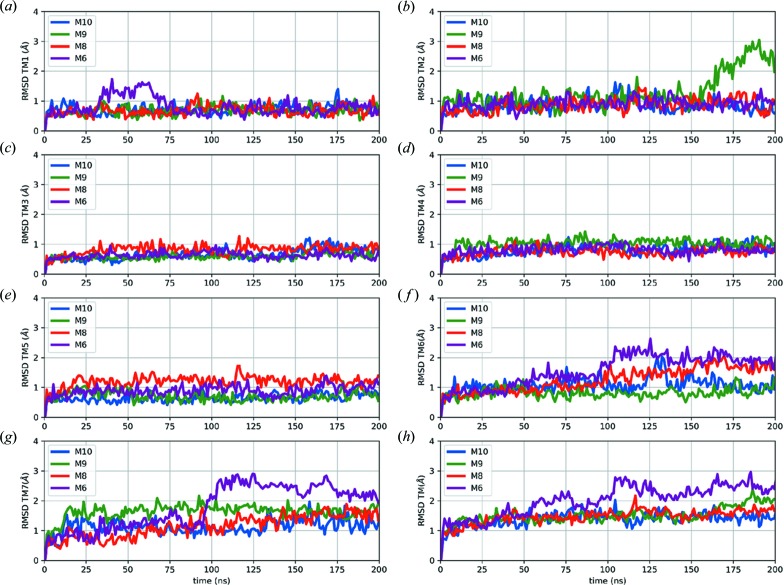
MD simulation results of the four crystallized constructs. (*a*)–(*g*) The RMSD of TMs 1–7 individually and (*h*) the RMSD of overall TMD. The RMSD was computed with respect to the equilibrated structures.

According to root-mean-square deviation (RMSD) analysis, the structure variations for T4L were within 2 Å compared with the initial structures for all four systems, indicating that the T4L domain was highly stable in all constructs (Fig. S3). While the TMDs of the three constructs (M10, M9 and M8) exhibited similar conformational fluctuations, the structural changes for TMs of M6 were relatively large (3 Å), indicating that the back mutations in M6 destabilized the TMD of the receptor (Figs. 3 ▸ and S3). The 2D RMSD cluster analysis revealed that the top five clusters of M6 covered only 60% of total populations during the 200 ns simulation, whereas in the other mutants, top five clusters could cover >80% of total populations (Fig. S4). These simulations suggest larger conformational fluctuations of M6 compared with the other three constructs, thus providing a possible explanation for the low diffraction quality of M6. The structural changes of M6 mainly occurred in TMs 6 and 7 [Figs. 3 ▸(*f*) and 3 ▸(*g*)], while TMs 1–5 [Figs. 3 ▸(*a*)–3(*e*)] remained stable throughout the simulation; this is seemingly in contrast with the fact that these mutations were located at TM2 (C193^2.63b^S/F196^2.66b^I), TM3 (C233^3.36b^M) and TM4 (A271^4.47b^S). However, the structure of GLP-1R revealed strong intrinsic interactions between TM1–5 and TM6–7, and these interactions were necessary for GPCR dynamics and for the signal communications between the extracellular and intracellular sides of GPCRs. Considering Phe196^2.66b^ for example [Fig. 2 ▸(*e*)], the hydrophobic network around Phe196^2.66b^ strengthened the stability of the TM2–ECL1–TM3 region and the rigidity could be expanded to the nearby ECL2 region through the conserved TM3–ECL2 di­sulfide bond (Cys226^3.29b^—Cys296^ECL2^). Furthermore, this hydrophobic network also interacted with TM1 through multiple inter­actions, including a hydrogen bond between Asp198^2.68b^ and Tyr145^1.40b^. Therefore, the I196^2.66b^F mutation indirectly stabilized the network in the TM1–TM7–TM6 region that has been suggested to function as the conformational switch (de Graaf *et al.*, 2017 ▸) of class B receptors.

## 3. Discussion   

The GPCR TMD is highly dynamic because different conformational states co-exist physiologically in the cell membrane. To solve a GPCR crystal structure one needs to unify the receptor into a single state. To accomplish that, a high-affinity GPCR ligand (agonist or antagonist) is usually added to the extracellular side to shift the equilibrium to either the active or inactive state. At the intracellular side, G protein/mini-G protein/nanobody has recently been used to stabilize the GPCR active conformation, which is especially suitable for cryoEM structural determination because the complex with a G protein/mini-G protein/nanobody increases molecular size. However, the current cryoEM method cannot be used for the determination of a crucial state of GPCR, *i.e.* the antagonist/NAM-stabilized inactive conformation. Hence, both crystallography and cryoEM have their own advantages and drawbacks, and they can complement each other in determining macromolecular structures and in understanding their physiological functions. In GPCR structural biology studies, more and more mutations have been introduced to improve the expression level and thermal stability. In some cases alanine-scanning mutagenesis was used to search for thermal-stabilizing point mutations (Kean *et al.*, 2015 ▸), while in other situations mutations were designed by homolog modeling and computational approaches, for example, the *CompoMug* program that employs sequence-based analysis, structural information and a derived machine-learning predictor (Popov *et al.*, 2018 ▸). We designed 98 mutations in total during construct optimization and selected ten thermal-stabilizing mutations to assist the crystallization of inactive GLP-1R in complex with NAMs. These design strategies may be applicable for structure determination of class B or other families of GPCRs.

In the GLP-1R, as well as previous class B CRF_1_R and GCGR structures, the inactive conformation is stabilized by the central polar network around TM3, 5, 6 and 7, as well as the binding of ligands (de Graaf *et al.*, 2017 ▸). Specifically, the conserved Asn^5.50b^ forms polar contacts with the backbone of Tyr^3.44b^ and/or Met/Leu^3.47b^ and Phe^5.51b^ that packs against the Pro^6.47b^XXGly^6.50b^ bulge in TM6. In the active GLP-1R and CTR structures (Zhang, Sun *et al.*, 2017 ▸; Liang *et al.*, 2017 ▸), the Pro^6.47b^XXGly^6.50b^ bulge is unwound and stabilized by hydrogen-bond interactions with Asn^5.50b^ (Leu^6.49b^ backbone) and Gln^7.49b^ and His^6.52b^ (Pro^6.47b^ backbone). In the inactive GLP-1R structure, the di­sulfide linkage between I317^5.47b^C and G361^6.50b^C locks the conformation of the bulge in TM6 and, therefore, neither unwinds from the bulge nor swings out of the intact TM6 are possible in the active conformation. The strategy of introducing di­sulfide bonds has previously been demonstrated as useful for crystallization of GPCRs, for example, in the lysophosphatidic acid receptor 1 (LPA1) structure to link the extracellular tips of TM5 and TM6 (Chrencik *et al.*, 2015 ▸), but to our knowledge it has not been utilized before to lock a GPCR into a specific conformation. We want to highlight that a di­sulfide linkage such as I317^5.47b^C—G361^6.50b^C may be especially suitable for class B receptors as this conserved glycine residue (only found in class B) is located precisely at the bulge region, and its helix-breaker feature usually provides flexibility for the equilibrium of different conformations. The present data show that without this di­sulfide, the protein is unstable and not crystallizable. In contrast, removal of unlinked cysteines, as in the M8 construct, only slightly affects the quality of crystal diffraction.

Alternatively, replacing glycine by non-glycine residues is also expected to provide rigidity for the transmembrane helices and thus increases thermal stability, *e.g.* G163^4.60^N in the CCR5–maraviroc structure (Tan *et al.*, 2013 ▸), G103^3.49^A in the GPR40–TAK875 structure (Srivastava *et al.*, 2014 ▸), G675^3.60^M in the mGlu5–mavoglurant structure (Doré *et al.*, 2014 ▸), G215^ECL2^A in the NTSR1–NTS^8–13^ structure (White *et al.*, 2012 ▸), G361^6.50b^A in the GLP-1R−peptide 5 structure (Jazayeri *et al.*, 2017 ▸) and, in our case, G318^5.48b^I in GLP-1R–NAM structures (Song *et al.*, 2017 ▸). Apart from in CCR5 and GLP-1R where the mutations were specifically designed, mutations in other receptors were chosen by a random screening method, and the conformational tendencies were not described well in these studies.

In addition, mutations can also be considered to fit the local hydrophobic or hydrophilic environment, for example, the S225^3.28b^A, I196^2.66b^F and K346^6.35b^A mutations described above, and the A233^6.33^D mutation applied in the structure determination of CCR5–maraviroc (Tan *et al.*, 2013 ▸). An atypical case is the S271^4.47b^A mutation which we included in our constructs to fit the hydrophobic lipid bilayer environment, a strategy rarely used before in the structure determination of GPCRs.

Finally, one unique feature in the crystallization of GLP-1R is the C347^6.36b^F mutation introduced at the binding interface with the NAMs. Designed based on the sequence alignment within class B GPCRs, this mutation was predicted to increase the binding area with the hydrophobic arms of the NAMs while not affecting the binding modes. Our previous assay showed that the mutation could enhance the inhibitory potency of the NAM, as well as the thermal stability of the NAM-bound receptor. As a control, the mutation does not affect the potency of GLP-1 from the orthosteric pathway (Song *et al.*, 2017 ▸). However, caution should be taken when making mutations on the ligand-binding interface, which might change the original binding pose. Thus, this method relies on accurate prediction of the ligand-binding interface.

In conclusion, we have summarized all 98 tested mutations in the structural determination of GLP-1R in complex with NAMs. We specifically investigated four back mutations in this study and confirmed that I196^2.66b^F and S271^4.47b^A contributed differently to the receptor thermal stability and crystallization probability, while the other two mutations, S193^2.63b^C and M233^3.36b^C, may be dispensable. The mutagenesis strategies we successfully applied for crystallization of GLP-1R include mutations to: lock the receptor in a specific conformation (*e.g.* I317^5.47b^C—G361^6.50b^C), increase intramolecular (*e.g.* S225^3.28b^A, K346^6.35b^A, I196^2.66b^F) and intermolecular interactions for crystal packing (*e.g.* S271^4.47b^A), enhance the rigidity of a transmembrane helix (*e.g.* G318^5.48b^I), and strengthen the ligand–receptor binding interface (*e.g.* C347^6.36b^F). Our mutagenesis data indicate that hydrophobic interactions, although less specific, are more flexible, making it easier to achieve the initial design. Conversely, more specific interactions such as di­sulfide and hydrogen bonds are more challenging to implement because of their strict restraints on bond geometries. The mutagenesis principle described above can be used not only to crystallize GPCRs and other membrane proteins, but also to lock proteins into specific conformations for functional or pharmacological studies.

## 4. Methods   

### 4.1. Mutation screening   

The double-cysteine mutation screening (Table S1) was based on a construct of the TMD that included a thermo-stabilized apocytochrome *b*_562_RIL (BRIL) at the N-terminus and a T4L inserted into the intracellular loop 2. Sixteen pairs of double-cysteine mutants were screened in the first round and purified with ligand NNC0640 to stabilize the receptor during purification and generate more monomeric proteins. In the second round, no ligand was included since the proteins were relatively stable compared with the first round. One pair, I317^5.47b^C—G361^6.50b^C, immediately stood out from the list and was included in the second round (mutations L245^3.48b^C—N320^5.50b^C were included in the second round erroneously). A second mutation, S193^2.63b^C—M233^3.36b^C, also helped protein homogeneity and was included in the final crystallization construct, though we found that this pair was not di­sulfide linked after solving the structure, which indicated that the two cysteines may function independently to improve the monodispersity or thermal stability at that stage of construct optimization.

The screening of single point mutations (Table S2) was conducted at different stages: the first 23 were from an earlier stage without double-cysteine mutations, while the remaining 17 mutations were conducted based on the two pairs of double-cysteine mutants. We made two mutations (1 and 2) to break the potential dimerization as suggested previously (Harikumar *et al.*, 2012 ▸). Afterwards, we made five mutations (3–7) to increase the binding interactions with the ligand NNC0640, and 11 mutations (8–18) to form covalent interactions with a modified NNC0640 ligand based on docking models of NNC0640. The mutations 8–15 were based on a model where the ligand was docked to the orthosteric pocket that was proven wrong according to a later GCGR–MK0893 structure. The remaining mutations (19–40) were designed to strengthen local interactions between corresponding residues by forming hydrogen bonds (salt bridges), hydrophobic interactions and so on. At earlier stages including double-cysteine screening, we mainly selected the mutations with improved homogeneities since thermal-stability data were not reliable for proteins with high proportions of aggregation, while at later stages we mainly considered the thermal-stability data. Protein-yield data usually correlated with the monodispersity data so they were only taken into consideration in special cases. For example, mutant G318^5.48b^I did not increase homogeneity but its protein yield was much improved so we included it in the crystallization construct. It is worth pointing out that in each round the values of monodispersity/yield were compared with the controls rather than the wild type. In rare cases we comprehensively considered all three characteristics. For example, mutant S389^7.44b^L showed better monodispersity compared with the control, whereas the thermal stability was significantly lower. Therefore, we did not include it in the M10 crystallization construct. Furthermore, at that stage we already had one mutation (C347^6.36b^F) in the ligand–receptor interface that helped thermal stability.

At mutation screening stages, we mainly used NNC0640 for co-purification and thermal-stability measurement because of its availability. Both NNC0640 and PF-06372222 were used in the previous co-crystallization with the M10 construct, while only PF-06372222 was used in the crystallization of the three new constructs (M9, M8, M6) in the current study since PF-06372222 generated higher resolution data than NNC0640 in the M10 construct.

### 4.2. Purification of new GLP-1R mutants   

The new GLP-1R constructs (M9, M8, M6) containing the same expression cassette as M10 were purified and crystallized as previously described (Song *et al.*, 2017 ▸). Briefly, the Bac-to-Bac Baculovirus System (Invitrogen) in *Spodoptera frugiperda* cells was used for expression. Cells were infected at a density of 2–3 × 10^6^ cells ml^−1^, grown at 27°C, and harvested 48 h after infection. After two washes of low-salt buffer and three washes of high-salt buffer, the cell membrane was solubilized in the presence of 200 µ*M* ligand (PF-06372222), 2 mg ml^−1^ iodo­acetamide and (EDTA)-free protease inhibitor cocktail (Roche). 1.0%(*w*/*v*) DDM (*n*-do­decyl-β-d-malto­pyran­oside, Affymetrix) and 0.2%(*w*/*v*) CHS (cholesteryl hemisuccinate, Sigma-Aldrich) were used for the solubilization, and the supernatant was then collected and incubated with TALON IMAC resin (Clontech, Palo Alto, USA) at 4°C, overnight. After washing, the resin was resuspended and treated with tobacco etch virus protease (TEV), and the receptor protein was harvested in the flow through with buffer [25 m*M* HEPES, pH 7.5, 500 m*M* NaCl, 5%(*v*/*v*) glycerol, 0.01%(*w*/*v*) DDM, 0.002%(*w*/*v*) CHS, 30 m*M* imidazole and 100 µ*M* PF-06372222]. The protein was concentrated to ∼30 mg ml^−1^ with a 100 kDa molecular weight cutoff concentrator.

### 4.3. Thermal-stability assay   

CPM dye {N-[4-(7-di­ethyl­amino-4-methyl-3-coumarinyl)phenyl]­male­imide} was dissolved in DMSO (di­methyl sulfoxide) at 4 mg ml^−1^ as stock solution and diluted 20 times in CPM buffer [25 m*M* HEPES, pH 7.5, 500 m*M* NaCl, 5%(*v*/*v*) glycerol, 0.01%(*w*/*v*) DDM, 0.002%(*w*/*v*) CHS] before use. 1 µl of diluted CPM was added to the same buffer with ∼0.5–2 µg receptor in a final volume of 50 µl. The thermal-denaturation assay was performed in a Rotor-gene real-time PCR cycler (Qiagen). The excitation wavelength was 365 nm and the emission wavelength was 460 nm. All assays were performed over a temperature range of 25–85°C. The stability data were processed with *GraphPad Prism*.

### 4.4. Crystallization, data collection and structure refinement   

The protein sample was reconstituted into lipidic cubic phase (LCP) by mixing 40% of ∼30 mg ml^−1^ protein with 60% lipid [10%(*w*/*w*) cholesterol, 90%(*w*/*w*) monoolein]. Crystallization trials were performed using a syringe lipid mixer and the protein–lipid mixture was dispensed in 40 nl drops onto glass sandwich plates and overlaid with 800 nl precipitant solution using an NT8 (Formulatrix). For M9, M8 and M6, crystals appeared after 1–2 weeks in 0.4–0.45 *M* ammonium acetate, 0.1 *M* sodium cacodylate, pH 6.2–6.6, 35–38% PEG 400, 3%(*w*/*v*) amino­hexanoic acid, and reached their full size (150 × 50 × 10 µm) within 2–3 weeks. Crystals were harvested directly from LCP using 50–150 µm micromounts (M2-L19-50/150, MiTeGen), flash frozen and stored in liquid nitrogen. Initial diffractions were tested at the Shanghai Synchrotron Radiation Facility in China.

X-ray diffraction data were collected at the SPring-8 beamline 41XU, Hyogo, Japan, using a Rayonix MX225HE detector (X-ray wavelength 1.0000 Å). The crystals were exposed with a 10 µm minibeam for 0.2 s and 0.2° oscillation per frame, and a rastering system was applied to find the best-diffracting parts of single crystals (Cherezov *et al.*, 2009 ▸). *XDS* (Kabsch, 2010 ▸) was used for integrating and scaling data from the best-diffracting crystals. M9, M8 and M6 structures were solved by molecular replacement with *Phaser* using previous GLP-1R (PDB entry 5vew; Song *et al.*, 2017 ▸) as the search model. The structure was refined iteratively with *Phenix* (Adams *et al.*, 2010 ▸; Liebschner *et al.*, 2019 ▸) with manual modification using *Coot* (Emsley *et al.*, 2010 ▸). Structures were checked by *MolProbity* (Chen *et al.*, 2010 ▸) and statistics are provided in Table 1 ▸.

### 4.5. MD simulation   

The missing residues in ECL2 (Trp203–Leu218) and ECL3 (Asp372–Arg380) of M10 were fixed by the *FREAD* server while the missing heavy atoms in the M10 structure were fixed by *tleap* in *Amber* (Case *et al.*, 2005 ▸; Choi & Deane, 2010 ▸). M10 also contains the ligand (PF-06372222) and lipids OLC and OLA. The *CHARMM*36 force field parameters of ligand (PF-06372222) and detergents OLC and OLA were generated by *CGenFF* (Vanommeslaeghe & MacKerell, 2012 ▸).

The *PPM* server was used to reorient the GLP-1R-T4L systems to ensure that the transmembrane domain of GLP-1R was well located in the POPC (1-palmitoyl–2-oleoyl-*sn*-*glycero*-3-phospho­choline) lipid bilayer (Lomize *et al.*, 2012 ▸). For each system, 200 ns MD simulations were performed. Before the production ran, the systems were equilibrated in a POPC lipid bilayer and solvated in a water box. The *CHARMM* graphical user interface was used to generate topology and the parameter files for the GLP-1R-T4L systems (Wu *et al.*, 2014 ▸). In addition to the protein complex, ∼255 POPC molecules, ∼42 000 water molecules (TIP3) and excess sodium chloride ions were added to maintain an ion concentration of 150 m*M*; thus, the entire system contained ∼160 000 atoms. The systems were modeled using the force parameters of *CHARMM*36. The NPT ensemble (constant particle number, pressure and temperature) MD simulations were generated using *GROMACS* 5.1.2 (Hess *et al.*, 2008 ▸). The *REDUCE* program in *Amber* was used to add hydrogens to the original PDB files and to determine the protonation state of histidine residues (Word *et al.*, 1999 ▸).

The initial energy minimizations were achieved using the steepest descent algorithm and this process was followed by two steps of equilibrations, *i.e.* 20 ns NVT (constant particle number, volume and temperature) dynamics with large restraint force constants and 40 ns NPT dynamics with gradually decreased restraint force constants. These restraints included harmonic restraints on heavy atoms of the protein and planar restraints to hold the position of lipid head groups of membranes along the *Z* axis. The system temperature was set to 303 K. Once all the equilibration steps were completed, the constraints were removed and each system propagated under constant temperature and pressure for 200 ns with a time step of 2 fs.

To evaluate the stabilities, the structure changes caused by mutation were quantified using the RMSD of the atomic position of the TMD of a receptor with respect to the equilibrated structures. For the stability of fusion partner T4L, the RMSD was computed using the transformation matrix obtained by aligning the TMD of the receptor. This described the changes in relative motion of the T4L. The *CPPTRAJ* package in *Amber* tools was used to compute the 2D RMSD of the ligand–receptor complex to check the overall stability of the complex structure (Case *et al.*, 2005 ▸). For the 2D RMSD case, RMSD was calculated between pairwise snapshots obtained from the 200 ns simulation trajectories. The RMS fluctuation of each residue in the fusion partner and receptor was calculated to compare the thermal fluctuations of the systems.

We carried out clustering analysis to group these mutant structures based on their similarity (Wang *et al.*, 2019 ▸). First, the pairwise RMSD was computed based on the TMD of GLP-1R, then the Javis–Patrick method was used for clustering. Secondly, an RMSD of 1.5 Å was used as the difference cutoff to define structure clusters. If the RMSD value was smaller than 1.5 Å, the two structures were considered to be in the same cluster. These clustering results were used to gauge the stability of the ligand–receptor complex. A new structure was joined to an existing cluster if at least one structure in the cluster shared three common neighbors with the new structure, where the neighbors were the ten most similar structures or all the structures within the RMSD cutoff of 1.5 Å. The clustering program used for this analysis was implemented in the *GROMACS* package. The size of each cluster was correlated to the stability of the corresponding cluster. The results are summarized in Fig. S2.

## Supplementary Material

PDB reference: thermal-stabilized (M9) human GLP-1 receptor transmembrane domain, 6kjv

PDB reference: thermal-stabilized (M8) human GLP-1 receptor transmembrane domain, 6kk1

PDB reference: thermal-stabilized (M6) human GLP-1 receptor transmembrane domain, 6kk7

Supporting information. DOI: 10.1107/S2052252519013496/tj5027sup1.pdf

## Figures and Tables

**Table 1:** Data-collection and refinement statistics of GLP-1R mutants

Data collection				
Construct	M10	M9	M8	M6
Mutations†	**S193C, I196F, M233C, S271A,** S225A, G318I, K346A, C347F, I317C—G361C	**S193C, I196F, S271A,** S225A, G318I, K346A, C347F, I317C—G361C	**I196F, S271A,** S225A, G318I, K346A, C347F, I317C—G361C	S225A, G318I, K346A, C347F, I317C—G361C
Number of crystals	25	17	18	15
Space group	*P*1	*P*1	*P*1	*P*1
Cell dimensions				
*a*, *b*, *c* (Å)	64.8, 66.4, 83.4	65.0, 68.3, 83.4	64.9, 67.4, 83.7	65.2, 71.1, 81.0
α, β, γ (°)	90.5, 90.2, 107.7	91.5, 90.3, 106.5	91.07, 90.10, 107.9	92.5, 92.6, 105.1
Total reflections	133127	74289	80566	43577
Unique reflections	34615	30099	25859	20302
Resolution (Å)‡	50.0–2.7 (2.85–2.7)	45.2–2.8 (2.95–2.80)	49.50–2.80 (2.95–2.80)	41.20–3.10 (3.27–3.10)
*R*_merge_	0.12 (0.51)	0.12 (0.57)	0.13 (0.40)	0.11 (0.46)
Mean *I*/σ(*I*)	6.2 (1.4)	5.3 (1.4)	5.3 (2.2)	4.6 (1.6)
Completeness (%)	95.2 (84.2)	88.8 (79.7)	77.9 (70.9)	79.9 (74.5)
Redundancy	3.8 (1.9)	2.5 (1.8)	3.1 (2.6)	2.1 (1.9)
CC_1/2_	0.99 (0.61)	0.98 (0.62)	0.98 (0.76)	0.99 (0.61)
				
Refinement				
Resolution (Å)	30.0–2.7	29.8–2.8	49.5–2.8	40.4–3.1
Number of reflections (test)	34567 (1743)	30036 (1324)	25796 (1136)	20218 (955)
*R*_work_/*R*_free_ (%)	22.8/24.6	24.7/28.0	24.4/29.0	25.7/30.3
Average protein *B* factor (Å^2^)	97	87.9	90.7	107.9
Number of atoms (A, B)				
Protein	3302, 3305	3302, 3305	3302, 3315	3300, 3290
Ligand	37, 37	37, 37	37, 37	37, 37
Lipid and other	96, 72	31, 27	4, 3	0, 0
RMS deviation				
Bond lengths (Å)	0.01	0.004	0.009	0.013
Bond angles (°)	0.90	0.73	1.441	1.557
Ramachandran plot (%)§				
Favored regions	94.0	95.7	95.0	91.6
Allowed regions	6.0	4.3	5.0	8.3
Disallowed regions	0.0	0.0	0.0	0.1
PDB entry	5vew ¶	6kjv	6kkl	6kk7

† Bold indicates the sites where we carried out back mutation in this study.

‡ The highest resolution shell is shown in parentheses.

§ As defined in *MolProbity*.

¶ This PDB entry has been reported previously (Song *et al.*, 2017 ▸).
