# Supplementary material for: Mutagenesis facilitated crystallization of GLP-1R
Source: IUCrJ. 2019 Oct 17;6(Pt 6):996–1006. doi: 10.1107/S2052252519013496 (PMC6830218; doi:10.1107/S2052252519013496)
Supplement: Supplementary file 1 [file m-06-00996-sup1.pdf]

IUCrJ

Volume 7 (2020)

Supporting information for article:

Mutagenesis facilitated crystallization of GLP-1R

Yueming Xu, Yuxia Wang, Yang Wang, Kaiwen Liu, Yao Peng, Deqiang Yao, Houchao Tao, Haiguang Liu and Gaojie Song

Table S1 The screening of double-cysteine mutants.

Mutations (1st round)	Monodispersity [#]	Yield [σ]	Mutations (2nd round)	Monodispersity	Yield
K288$^{4.64b}$C-N304^{ECL2}C	0.992	0.954	L245$^{3.48b}$C-N320$^{5.50b}$C/A162$^{1.57b}$C-C403$^{7.58b}$	0.493	0.400
W284$^{4.60b}$C-L307$^{5.37b}$C	0.612	0.267	L245$^{3.48b}$C-N320$^{5.50b}$C/L189$^{2.59b}$C-C236$^{3.39b}$	0.678	0.406
W284$^{4.60b}$C-I308$^{5.38b}$C	0.790	0.504	L245$^{3.48b}$C-N320$^{5.50b}$C/A238$^{3.41b}$C-V281$^{4.57b}$C	0.808	0.710
V281$^{4.60b}$ C-L311$^{5.41b}$C	0.982	0.558	L245$^{3.48b}$C-N320$^{5.50b}$C/L255$^{3.58b}$C-K346$^{6.35b}$C	0.766	0.610
F280$^{4.56b}$C-L311$^{5.41b}$ C	0.794	0.514	L245$^{3.48b}$C-N320$^{5.50b}$C/E139$^{1.34b}$C-A208^{ECL1}C*	N/A	N/A
L245$^{3.48b}$C-N320$^{5.50b}$C	0.532	0.235	**I317$^{5.47b}$C-G361$^{6.50b}$C/S193$^{2.63b}$C-M233$^{3.36b}$C**	**1.169**	**1.514**
A256$^{3.59b}$C-I330$^{5.60b}$C	0.969	0.731	I317$^{5.47b}$C-G361$^{6.50b}$C/S186$^{2.56b}$C-A239$^{3.42b}$C	0.719	0.274
A200$^{2.70b}$C-D222^{ECL1}C	0.882	0.975	I317$^{5.47b}$C-G361$^{6.50b}$C/F187$^{2.57b}$C-G395$^{7.50b}$C	0.349	0.105
L189$^{2.59b}$C-C236$^{3.39b}$*	N/A	N/A	I317$^{5.47b}$C-G361$^{6.50b}$C/F156$^{1.51b}$C-A191$^{2.61b}$C	0.869	0.495
A200$^{2.70b}$ C-S225$^{3.28b}$C	0.909	0.779	I317$^{5.47b}$C-G361$^{6.50b}$C/G285$^{4.61b}$C-I308$^{5.38b}$C	0.876	0.712
**I317$^{5.47b}$C-G361$^{6.50b}$C**	**1.273**	**1.816**	I317$^{5.47b}$C-G361$^{6.50b}$C/G248$^{3.51b}$C-T353$^{6.42b}$C	0.457	0.146
I317$^{5.47b}$C-V365$^{6.54b}$C	0.560	0.344	I317$^{5.47b}$C-G361$^{6.50b}$C/A239$^{3.42b}$C-P277$^{4.53b}$C	0.503	0.152
N320$^{5.50b}$C-I357$^{6.46b}$C	0.346	0.127			
A162$^{1.57b}$C-C403$^{7.58b}$	0.479	0.276			
E139$^{1.34b}$C-A208^{ECL1}C	0.529	0.318			
A238$^{3.41b}$C-V281$^{4.57b}$C	0.919	0.631			
L255$^{3.58b}$C-K346$^{6.35b}$C	1.002	1.214			

* Cloning failed and no protein was expressed.

[#] Monodispersity: the percentage of monomeric fraction in total fractions in SEC. The data were calculated by dividing the values of mutants by the values of controls.

[σ] Yield: the heights of mutants' monomeric fractions divided by the values of controls in SEC. Note: we did not take into account the aggregation fractions. Therefore, this value may not represent the expression level of mutants since the purification result correlates with both expression level and extraction efficiency from the membrane. The latter usually reflects protein stability and sometimes it is more important for purification.

Color codes: >115% control (green); 85%-115% (white); 55%-85% (yellow); <55% (red).

Bold indicates the mutations that included in the final crystallization construct.

Table S2 The screening of single point mutants.

	Mutation	Rational	Monodispersity#	Yieldσ	TmΦ
1	Y269$^{4.45b}$A	To break the potential dimerization	0.879	0.967	
2	S271$^{4.47b}$A	To break the potential dimerization	1.250	1.378	
3	L141$^{1.36b}$Y	Introducing aromatic residues or N-glycans within the orthosteric pocket to increase ligand-binding (NNC0640 was wrongly predicted to bind to this pocket) interface	1.110	1.269	
4	L201$^{2.71b}$Y		0.885	1.223	
5	H374^{ECL3}Y		0.655	0.831	
6	R299^{ECL2}N		0.714	1.149	
7	N302^{ECL2}T		0.772	1.103	
8	L141$^{1.36b}$C	To form covalent interaction with the electrophile group of modified NNC0640 (these mutations are based on a wrong model of GLP-1R in which the ligand NNC0640 was docked to the orthosteric pocket)	0.926	0.641	
9	L142$^{1.37b}$C		0.980	0.940	
10	L144$^{1.39b}$C		0.984	0.346	
11	W203^{ECL1}C		0.958	0.521	
12	V237$^{3.40b}$C		0.930	0.377	
13	I309$^{5.39b}$C		0.451	0.223	
14	F385$^{7.40b}$C		0.799	0.511	
15	S389$^{7.44b}$C		0.955	0.648	
16	V332$^{5.62b}$C	To form covalent interaction with the electrophile group of a modified NNC0640 (mutations are based on the model of GCGR-MK0893 in which the ligand binds to the outside of TM6)	0.909	1.051	
17	S352$^{6.41b}$C		0.895	0.965	
18	L359$^{6.48b}$C *		N/A	N/A	
19	S301^{ECL2}R	Predicted to form hydrogen bond with E292$^{4.68b}$.	0.771	0.733	
20	A208^{ECL1}K	To form hydrogen bond with E139$^{1.34b}$ to stabilize ECL1-TM1	0.972	1.411	
21	A208^{ECL1}R	To form hydrogen bond with E139$^{1.34b}$ to stabilize ECL1-TM1	0.900	0.980	
22	M340^{ICL3}F	To strengthen the hydrophobic TM5-TM6 interactions	0.856	0.687	
23	M340^{ICL3}Y	To strengthen the hydrophobic TM5-TM6 interactions	0.596	0.374	

24	G151$^{1.46b}$A	To strengthen the helical conformation of TM4	0.758	0.326	-1.74
25	S163$^{1.58b}$L	To fit the local hydrophobic environment	0.654	0.490	-3.45
26	C174$^{2.44b}$S	To form hydrogen bond with E408	0.718	0.884	-0.05
27	**G318$^{5.48b}$I**	To strengthen the helical conformation of TM6	0.821	1.362	0.2
28	G248$^{3.51b}$S	To form hydrogen bond with N320$^{5.50b}$	0.784	1.127	-0.49
29	G275$^{4.51b}$A	To strengthen the helical conformation of TM5	0.747	0.910	-2.42
30	N320$^{5.50b}$L	To fit the local hydrophobic environment	0.968	1.149	-0.91
31	T343^{ICL3}E	To form hydrogen bond with nearby R348$^{6.37b}$ or N407$^{8.48b}$	0.881	1.218	0.43
32	R176$^{2.46b}$A	R176$^{2.46b}$A was proved to favor an inactive conformation	0.910	1.261	-3.36
33	**S225$^{3.28b}$A**	To fit the nearby hydrophobic environment	1.032	1.297	0.68
34	**I196$^{2.66b}$F**	To fit the nearby hydrophobic environment	1.031	1.007	0.30
35	**K346$^{6.35b}$A**	To fit nearby residues L254$^{3.57b}$, L255$^{3.58b}$ and K334$^{5.64b}$	1.08	1.629	0.08
36	K346$^{6.35b}$V	To fit nearby residues L254$^{3.57b}$, L255$^{3.58b}$ and K334$^{5.64b}$	0.95	0.856	-0.98
37	E364$^{6.53b}$F	Experimentally tested to increase inhibitory potency of NAMs	0.840	0.840	-1.55
38	**C347$^{6.36b}$F**	To increase hydrophobic interface with NAMs	1.063	1.212	2.14
39	L401$^{7.56b}$F	Predicted to increase hydrophobic interaction with NAMs	0.950	0.912	-0.89
40	S389$^{7.44b}$L	To fit local aromatic residues F390$^{7.45b}$ and F393$^{7.48b}$	1.050	1.190	-1.82

[*, #, σ] See Supplementary Table S1.

[Φ]Tm (melting temperature) values were measured with ligand NNC0640 and are shown for constructs 24-40 that include two pairs of double-cysteine mutants. These samples were relatively stable and the thermal stabilities are well reflected by the Tm. For the first 23 mutations, the proteins were quite unstable with high proportions of aggregations. Therefore, the data were not reliable and not presented here. Tm values were relative to controls and absolute values for representative mutations are shown in Fig. S1.

Monodispersity and yield color codes: >100% of control (green); 85%-100% (white); 55%-85% (yellow); <55% (red).

Tm color codes: mutations with increased and decreased values compared to controls are marked in green and yellow, respectively.

Bold indicates the mutations that included in the final crystallization construct.

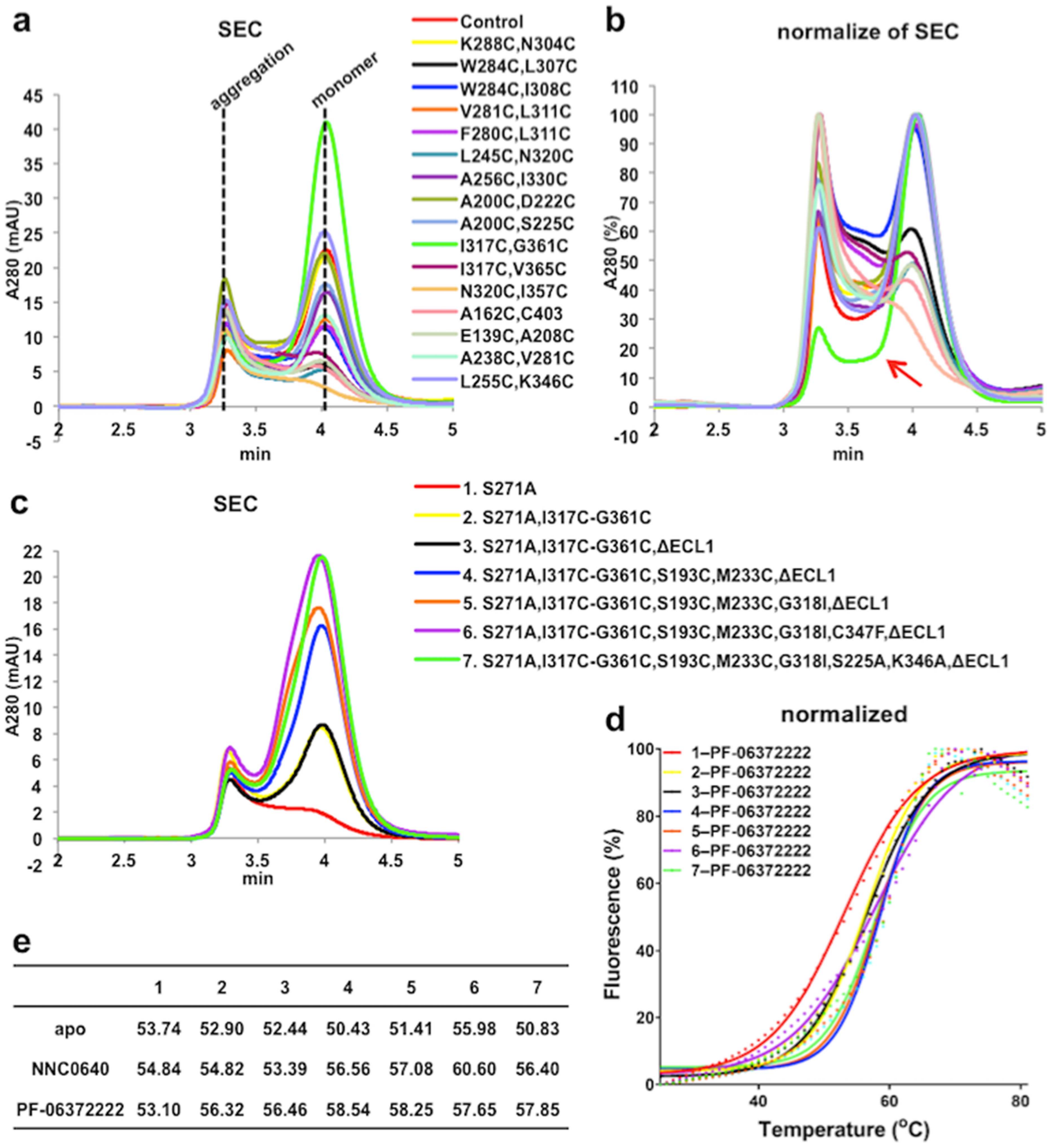

e

	1	2	3	4	5	6	7
apo	53.74	52.90	52.44	50.43	51.41	55.98	50.83
NNC0640	54.84	54.82	53.39	56.56	57.08	60.60	56.40
PF-06372222	53.10	56.32	56.46	58.54	58.25	57.65	57.85

Figure S1 Representative results of mutation screening. a-b, SEC and normalized SEC of first round of double-cysteine mutants. The aggregation and monomeric fractions are indicated with dash lines. The profile of mutant I317$^{5.47b}$C-G361$^{6.50b}$C is indicated with a red arrow; c, SEC profiles of representative constructs of double-cysteine and single point mutants during construct optimization. In samples 3-7, 10 residues from ECL1 (204-213) were replaced for crystallization and the truncations did not affect the SEC profiles; d-e, CPM profiles (with PF-06372222) and statistics (apo/NNC0640/PF-06372222) of the same representative constructs as in c.

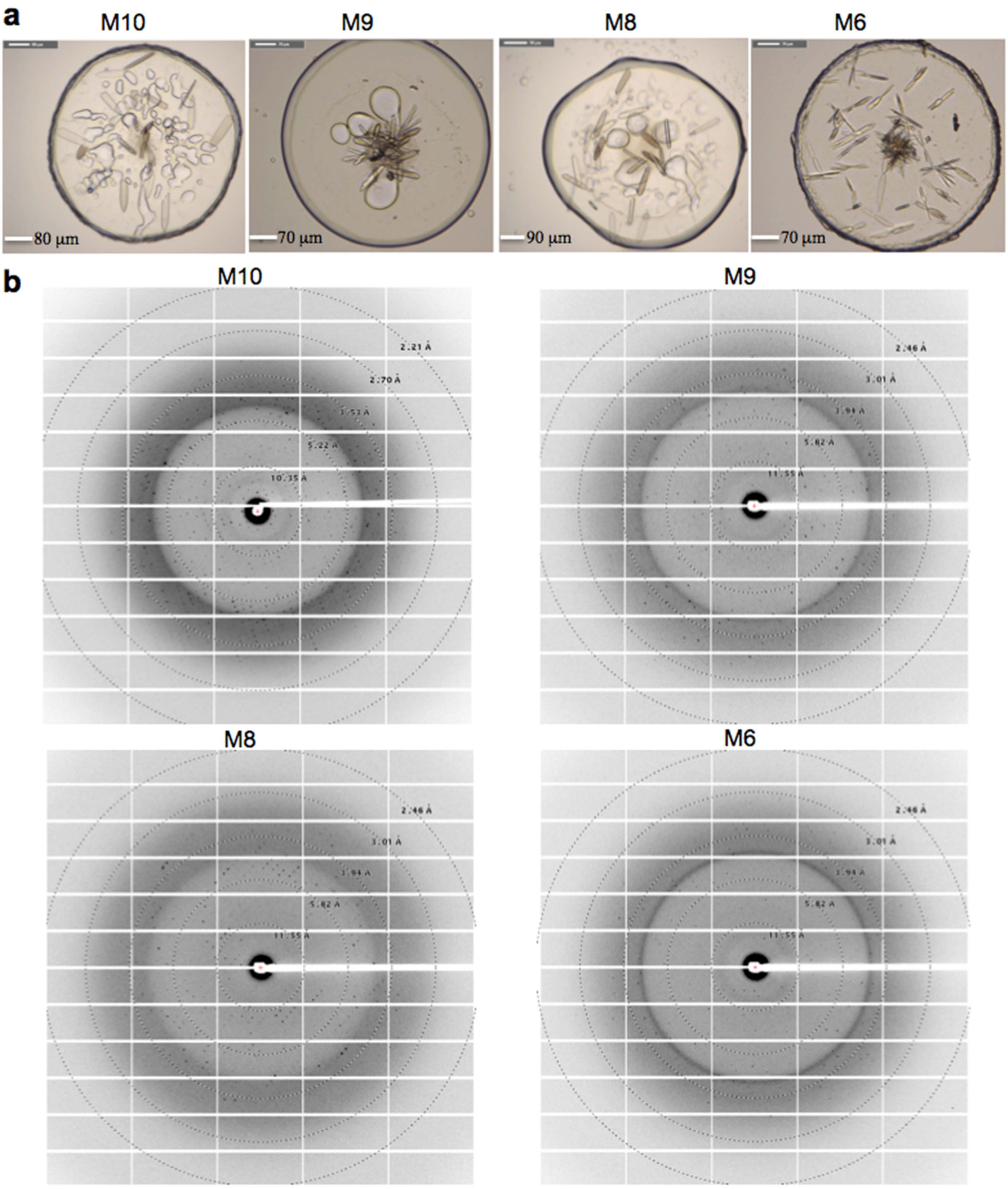

Figure S2 Representative crystals (a) and diffraction patterns (b) of the crystallized GLP-1R mutants.

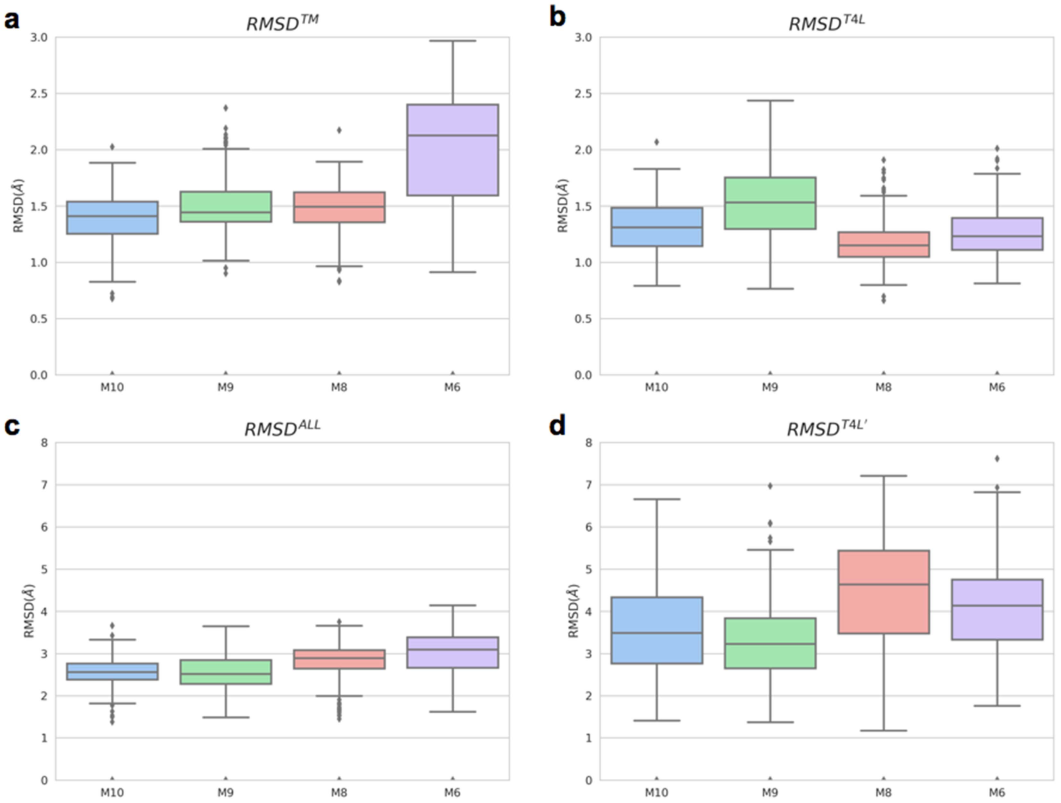

Figure S3 **Statistics of RMSD.** The plots are shown in box representation, with the middle 50% represented by the box, and the standard deviations shown as error bars. a, RMSD of GLP1R TMDs; b, RMSD of T4L domain; c, RMSD of TMD together with T4L; d, Relative RMSD of T4L by optimizing the alignment of the TMD of GLP1R. Both the RMSD for TMD and the overall RMSD suggest that the M6 is the least stable construct.

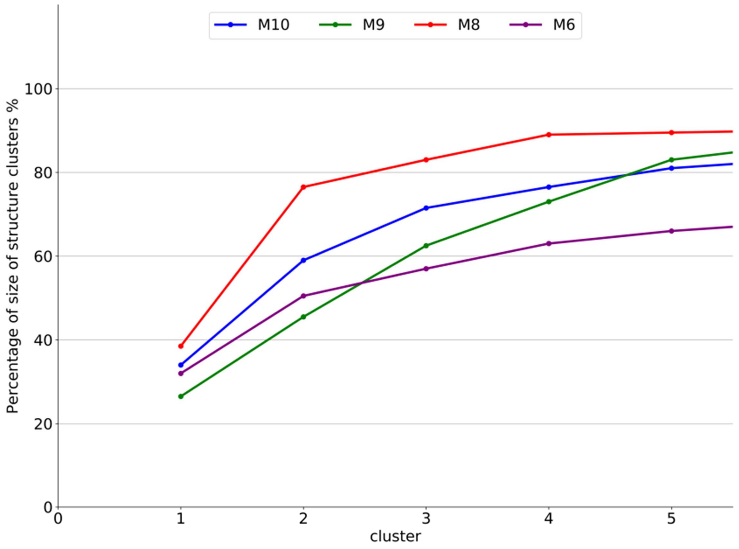

Figure S4 **Clustering results based on the pairwise RMSD values.** In M10/M9/M8, larger clusters (>80% populations clustered into top five clusters) correspond to deeper energy minimum, which resulted in higher energy barrier for the transitions between different conformations. In contrast, the M6 construct may have a smooth energy landscape with lower energy barriers, so the transitions between different conformational states occur easily.
